# Influence of physical activity on periodontal health in patients with type 2 diabetes mellitus. A blinded, randomized, controlled trial

**DOI:** 10.1007/s00784-021-03908-6

**Published:** 2021-04-01

**Authors:** K. Wernicke, J. Grischke, M. Stiesch, S. Zeissler, K. Krüger, P. Bauer, A. Hillebrecht, J. Eberhard

**Affiliations:** 1 Hannover Medical School, Hanover, Germany; 2 Department of Prosthetic Dentistry and Biomedical Materials Science, Hannover Medical School, Carl-Neuberg-Str.1, 30625 Hannover, Germany; 3 Sportpark Zwickau, Zwickau, Germany; 4 Justus-Liebig-Universität Gießen, Gießen, Germany; 5 Volkswagen Nutzfahrzeuge, Hanover, Germany; 6 The University of Sydney School of Dentistry and the Charles Perkins Centre, Faculty of Health and Medicine, The University of Sydney, Sydney, Australia

**Keywords:** Diabetes, Periodontitis, Physical activity, HbA1c, Periodontal health

## Abstract

**Objectives:**

The aim was to investigate the effect of physical activity on periodontal health and HbA1c levels in patients with type 2 diabetes mellitus (T2DM) over a period of 6 months.

**Materials and methods:**

Thirty-seven patients with non-insulin-dependent T2DM were included in the study. The intervention group (*n*=20) performed physical activity over a period of 6 months. The control group (*n*=17) did not receive any intervention. Baseline and final examinations included dental parameters and concentrations of glycosylated hemoglobin (HbA1c) and high-sensitivity C-reactive protein (hsCRP).

**Results:**

Physical activity showed a positive effect on periodontal health. Both the BOP (*p*= 0.005) and the severity of periodontitis (*p*= 0.001) were significantly reduced in the intervention group compared to the control group. Furthermore, HbA1c levels were reduced (*p*= 0.010) significantly in the intervention group while hsCRP levels significantly increased in the control group (*p*= 0.04).

**Conclusions:**

Within the limitations of this randomized, controlled trial, physical activity over a period of 6 months is a health-promoting measure for patients with T2DM and improves both periodontal health and HbA1c concentrations.

## Introduction

Diabetes mellitus is one of the most common chronic metabolic diseases and results from an absolute insulin deficiency, insulin resistance, or defective insulin production. Type 2 diabetes mellitus (T2DM) is the most common chronic disease in the Western world. Already in the 1970s, studies found that T2DM patients were more likely to develop periodontal diseases than healthy people [1].

Diabetes has been identified as an important risk factor for periodontitis. The risk of developing periodontitis and peri-implantitis is significantly increased in patients with diabetes compared to healthy control groups [2–5]. This phenomenon was described in various studies and a causal connection between T2DM and periodontitis was postulated [6–8]. In a clinical study, a significant reduction in blood glucose (HbA1c values) was found after non-surgical periodontal therapy in patients with moderate to advanced chronic periodontitis [9, 10]. However, the exact causal mechanism has not yet been conclusively investigated. Possible links between diabetes and chronic periodontitis may include local and systemic inflammatory responses, the presence of advanced glycation end products (AGEs), oxidative stress, or mitochondrial dysfunction [11, 12]. In addition, pathogenic bacteria and their by-products induce the synthesis of acute phase proteins, cytokines, and oxidative stress molecules in the liver, which subsequently reduce insulin sensitivity [7].

Studies including lifestyle and health interventions have shown that they have a positive health effect on patients with T2DM. For example, physical activity has been shown to improve the metabolic condition of T2DM patients, reduce HbA1c levels and cardiovascular mortality, and improve the quality of life, lipid levels, and blood pressure [13, 14]. Periodontal health has also been significantly improved by physical activity in clinical trials [15, 16]. Other studies found significantly lower plaque indices, gingival indices, and less clinical attachment loss in physically active subjects compared to less active subjects [17]. At the molecular level, reactive oxygen species (ROS) may play a role as a link between physical activity, T2DM, and periodontitis. In clinical studies, systemic ROS levels were reduced by physical activity, while chronic periodontitis had the opposite effect [18, 19]. It was also shown that high systemic ROS concentrations are associated with decreased glycemic control [20].

To test the hypothesis that physical activity is a health-promoting measure with significant positive effects on periodontal health and HbA1c concentrations, this examiner-blinded, controlled clinical trial was conceived in subjects with non-insulin-dependent T2DM over a period of 6 months.

## Material and methods

### Study population

Participants >18 years old with non-insulin-dependent type 2 diabetes mellitus were recruited by information sessions in a gymnastic hall and advertisements at regional doctors in a medium-sized town in Germany (Zwickau, Saxony, Germany). The study aimed for the inclusion of a representative sample with respect to sex, age, and ethnicity. Informed oral and written consent was obtained from each participant.

### Inclusion and exclusion criteria

This randomized clinical trial has been carried out in accordance with The Code of Ethics of the World Medical Association (Declaration of Helsinki) and was approved by the local Ethics Committee of the Justus-Liebig University Giessen, Germany (reference number 2011–0006). The study was performed by the Department of Sports Medicine of the University of Giessen, Germany, in collaboration with Hannover Medical School, Germany. The trial was registered at ClinicalTrials.gov (NCT 01377558).

T2DM was defined according to the WHO as described in [21]. Inclusion criteria were non-insulin-dependent T2DM and the willingness to participate in a baseline and follow-up dental examination. Exclusion criteria were unstable coronary artery disease, any serious medical condition that prevented adherence to the study protocol, or the ability to exercise safely, advanced retinopathy, and current insulin therapy. Furthermore, patients with preexisting physical activity of ≥ 60 min per week were not eligible.

### Exercise program

We conducted a 26-week, single center, randomized, controlled trial with a parallel group design. Previously inactive persons with type 2 diabetes were randomly assigned to 1 of 4 groups: aerobic exercise, resistance training, combined aerobic and resistance training, or a control group that reverted to pre-study exercise levels.

Altogether, 126 patients with type 2 diabetes were willing to participate. They were randomly assigned and matched 1:1 to the four study groups. After exclusion of 16 patients as screening failure, 110 patients remained. Thirty patients were assigned to the aerobic exercise group (group AE), 27 to the resistance-training group (RE), 25 to the combined training group (CE), and 28 patients to the control group (CG).

All participants were provided with a 6-month membership at an exercise facility. The membership fees were covered by the study funding to remove economic barriers to participation. Individual exercise supervision was provided with a fitness coach twice a week throughout the study. Attendance was verified through direct observation, exercise logs, and individual smart card controllers. Participants with >10% missed trainings were excluded.

The exercise was carried out twice a week for 6 months. Each exercise unit was preceded by a 10-min warm up period. The training duration and intensity gradually progressed after four and 13 weeks, respectively. The intervention groups exercised according to the study protocol of the assigned group. All participants in the intervention group took regularly part in the training.

The training method was based on published programs of the working group [22]. Intervention group 1 (strength endurance) completed a strength-endurance training after a general warm up and stretching in a group setting, followed by two passes of a strength-endurance circuit. The strength-endurance training consisted of eight machine-supported exercises that included all major muscle groups (leg abduction, leg adduction, back extension (lower back), dips, vertical row, vertical traction, leg press, abdominal crunch) and were performed for 1 min each. During the initial training session, a maximum force test with three attempts was performed. The best of the three tests was scored and used to define the exercise intensity at about 60% of the participant’s maximum force for the first 2 weeks of training. For the subsequent weeks, the load was increased by 10% and again by 5% for the last 4 weeks. Intervention group 2 (endurance) completed a progressive endurance training 2 times a week. After a general warm up and stretching in a group setting a 30-min training on a treadmill (technogym run 500/technogym run 600) or a bicycle ergometer with or without backrest (lifefitness-lifecycle 9500 HR) was performed. Intervention group 3 (combination intervention) completed a training combining both programs: one time a week an endurance and one time a week a strength endurance training was performed. All participants of the intervention groups regularly participated in the training.

Participation, correct exercise performance and execution, and the compliance of the participants were supervised by professional trainers under supervision of a sport scientist with doctors’ degree. All trainings took place in a certified sports center (certified by AG Diabetes, Sport und Bewegung der deutschen Gesellschaft für Geriatrie DGG).

All participants were advised not to change eating habits or to start diets during the study. Additionally, we took steps to minimize medication co-interventions by sending letters to participants’ physicians to inform about the study. We asked to maintain any antihypertensive and lipid-lowering therapy during the 6 months exercise intervention period. Adaption of medical therapy was possible for medical reasons. Changes in glucose-lowering medication should be reported either by the participant or the physician to the study group. We did not initiate any changes in medication throughout the study and devices used during the study are depicted in Table 1. The control group did not receive a sports program or any other lifestyle intervention.

**Table 1:** An overview over the sport equipment that was accessible to the participants of the intervention group over a period of 6 months. The left table column summarizes the strength endurance equipment and the right column the cardio endurance devices

Strength endurance devices (milon strength endurance circle)	Cardio endurance devices
Leg abduction	Treadmill (technogym run 500/technogym run 600)
Leg adduction	
Back extension (lower back)	Bicycle with backrest (lifefitness-lifecycle 9500 HR)
Dips-vertical row-vertical traction	Bicycle without backrest (lifefitness-lifecycle 9500 HR)
Leg press	
Abdominal crunch	

### Medical and dental records

A qualified dentist interviewed the participants on pre-existing periodontal diseases and habits including smoking and frequencies of dental check-ups. Body mass index (BMI) and weight in kilograms were recorded at baseline and after 6 months. Furthermore, any history of periodontitis was recorded.

### Assessment of periodontal conditions

For each patient, periodontal probing depth (PPD), bleeding on probing (BOP), and plaque scores were measured using a Florida probe system. PPD measurements were done at 6 sites per tooth (mesio-buccal, buccal, disto-buccal, mesio-oral, oral, and disto-oral) and BOP were recorded at 4 sites (mesio-buccal, disto-buccal, buccal, and oral). Furthermore, a periodontal anamnesis collected data on history of periodontitis. Periodontitis was classified according to Eke and Page et al. [23]. However, recently, a new international classification has been published. Severity of periodontal disease according to Eke and Page et al. (healthy, light, moderate, and severe) were denoted as (modified) stages (1, 2, 3, 4) according to Papapanou et al. (2018) without including a grading [24]. Full-mouth plaque index (API) was measured in % [25].

### Blood sampling

In addition to the dental examination, blood samples of each patient were collected in the morning after fasting and hsCRP and HbA1c levels were measured in mg/L or % at baseline and after 6 months. Blood analyses were performed in a certified clinical laboratory (Laboratory Community Saxony West, Zwickau, Germany) using the Blood Analyses Modular P800 (Roche-Institut). HbA1c levels were measured using an Adams A1c HA8180V Analyzer (Axon Lab AG, Reichenbach, Deutschland).

### Statistical analysis

All data were electronically stored. The statistical analysis was carried out in Excel (Version 2102, Microsoft, USA). Due to the small number of patients in the sport intervention groups, all three sports interventions were pooled as the intervention group and compared with the control group irrespective of the training method. First, *t* tests with equal variances were calculated to compare changes between baseline and the 6-month follow-up for participants’ weight, BMI, HbA1c, hsCRP, BOP, and periodontal staging between the sport intervention and the control group. Second, a multivariate regression analysis was performed to identify predictors of for the clinical parameter BOP as a marker of acute periodontal inflammation. The significance level was set at *P*<0.05.

## Results

A total number of 108 participants (women *n*=65, men *n*=43, age range 46 to 73 years) met the inclusion criteria and were randomized to the physical exercise or control groups. Thirty-one patients were excluded after the start of the study because patients did have no teeth (*n*=5) or refused a dental examination (*n*=26) leaving 77 at the baseline assessment. Thirty-seven participants were available for the final assessment with 20 participants of the physical activity group and 17 patients of the control group (Table 2). The mean HbA1c for all participants was 6.7% with a range between 5.6 and 9%. A mean BMI of 32.2 with a range between 21.9 and 46.1 was calculated. The mean hsCRP concentration was 0.86 mg/L with a range between 0.1 and 6.1 mg/L at baseline.

**Table 2:** The results (mean values ± standard deviations) of all participants who received dental examination at baseline and a final examination

	Control (*N*= 17)		Intervention (*N*= 20)	
	Baseline	Final	Baseline	Final
hbA1c (%)	6.64 ± 0.57	7.24 ± 0.79	6.71 ± 0.79	6.66 ± 0.70
hsCRP (mg/l)	0.72 ± 0.71	2.35 ± 3.62	1.01 ± 1.380	1.03 ± 1.51
BMI (kg/m^2^)	31.86 ± 6.68	33.11 ± 6.92	32.41 ± 4.39	31.34 ± 4.61
BOP (#sites)	1.35 ± 1.88	3.41 ± 6.19	14.5 ± 19.84	2.4 ± 4.09
Staging	1,29 ± 0.46	1,53 ± 0.50	1.55 ± 0.50	1.1 ± 0.3
PPD (mm)	1.41 ± 0.50	1.44 ± 0.54	2.27 ± 0.60	1.38 ± 0.28
Plaque (%)	21.64 ± 21.16	12.76 ± 15.24	26.90 ± 27.68	32.5 ± 26.39
Weight (kg)	92.24 ± 20.10	96.03 ± 19.64	92.47 ± 14.93	89.33 ± 15.74

### Univariate analysis

No significant differences between the intervention and control group for changes between baseline and final assessment were found for weight (*P*= 0.103), BMI (*P*= 0.144), and plaque index (*P*= 0.06). Significant differences were found for HbA1c concentration (*P*= 0.011), hs CRP concentration (*P*= 0.040), BOP (*P*= 0.002), PPD (*P*= <0.001), and periodontal staging (*P*= <0.001). HbA1c levels, BOP, PPD, and periodontal staging significantly improved in the intervention compared to the control group. The hsCRP concentrations increased significantly in the control compared to the intervention group.

### Multivariate analysis

In the multivariate analysis, all parameters were initially included in a statistical model (Tables 3 and 4) to identify independent risk factors for the variables BOP, periodontal staging, and HbA1c concentrations.

**Table 3:** Multivariate regression analysis with (modified) periodontal staging after intervention as primary outcome. The significant results are printed in bold. The modified staging is significantly associated with the intervention group. History of periodontitis is an independent risk factor for more severe periodontitis. *CRP* C-reactive protein, *BMI* body mass index, *HbA1c* glycated hemoglobin, *BOP* bleeding on probing, *staging* classification into the periodontal severity according to Papapanou Sanz et al. (2018)

	*t* statistic	*p* value
CRP (final)	0.315	0.755
HbA1c in % (final)	0.131	0.896
Intervention group	3.757	**<0.001**
Age	0.874	0.389
Gender	1.567	0.129
Smoking status	0.012	0.990
BMI in kg/m^2^ (final)	1.921	0.066
Plaque index (final)	1.085	0.287
BOP (final)	1.675	0.106
Recall frequency	0.262	0.795
History of periodontitis	2.444	**0.021**

**Table 4:** Multivariate regression with BOP after 6 months as primary outcome variable. The significant results are printed in bold. Lack of physical activity and the plaque index are independent risk factors for BOP. *CRP* high sensitivity C-reactive protein, *BMI* body mass index, *HbA1c* glycated hemoglobin, *BOP* bleeding on probing, *staging* classification into the periodontal severity according to Papapanou Sanz et al. (2018)

CRP (final)	0.507	0.616
HbA1c in % (final)	1.582	0.126
Intervention group	4.133	**<0.001**
Age	0.513	0.611
Gender	0.477	0.637
Smoking status	1.065	0.297
BMI in kg/m^2^ (final)	1.119	0.273
Plaque index (final)	5.323	**<0.001**
History of periodontitis	1.555	0.132
Recall frequency	1.353	0.187
PPD (final)	1.211	0.236

A multivariate regression analyses with HbA1c as the outcome variable showed that HbA1c improved significantly in study participants who participated in a sports intervention (*p* = 0.037) compared to control.

## Discussion

The present study investigated whether a sports intervention over a period of 6 months is an oral and general health-promoting measure in patients with T2DM. The results showed that a sports intervention of at least 6 months in patients with T2DM has a significant positive effect on periodontal health and HbA1c concentrations compared to a control group without sports intervention. The comparison of the sports intervention group with the control group showed a significant reduction in BOP, a significant improvement in the severity of periodontal disease, and significantly lower HbA1c levels in the intervention group at the final examination.

These results are consistent with the results of recent studies that have shown that physical activity is associated with a reduced prevalence of periodontitis and improved periodontal health of the population [26–28]. A study by Su-Jin Han et al. [27] showed a significant association between regular walking and a lower prevalence of periodontitis. Merchant et al. [28] confirmed these results. However, these studies have investigated the association between periodontitis and physical activity in a population without T2DM; to the knowledge of the authors, a study including patients at risk for T2DM has not been published yet.

The present study showed that physical activity was associated with improved periodontal health in patients with T2DM. However, a causal link between periodontitis, T2DM, and physical activity was not investigated. In the future, predictive models and individualized treatment options may be developed for patients with T2DM and periodontitis with the help of artificial intelligence and machine learning [29].

The significant reduction of BOP and the improvement of PA staging in the sports intervention groups may indicate that inflammatory processes in the oral cavity can be reduced by regular physical activity. However, periodontitis history and the plaque index were identified as independent predictors of BOP and the severity of periodontal disease as well.

Periodontitis and peri-implantitis are characterized by an inflammatory reaction, triggered by the host response to the pathologic oral microbiota with destruction of tissues [3, 30, 31]. Health-promoting measures such as brushing teeth, flossing, healthy nutrition, and regular dental examinations are important to prevent or contain the development and progression of oral infectious disease [15, 32, 33]. In line with the results of the current study, other clinical studies have already shown that physical activity is considered a health-promoting measure and even leads to a reduction in the prevalence of periodontitis in the general population [15]. This reduction in prevalence could be related to the effects of physical activity on cytokine production and immune modulation [34–36]. Studies in patients with cardiovascular disease have shown that physical activity modulates multiple cytokines, in particular CRP, which is a by-product of the liver metabolism and plays an key role in the acute phase response. An elevated level of this inflammatory biomarker is associated with diabetes. Recent studies found an association between increased CRP levels in patients with periodontitis and other systemic conditions such as coronary heart disease, stroke, or diabetes mellitus [37, 38]. Not only CRP but also interleukin 1 beta (IL-1) is related to the inflammatory response of several disease [36, 39]. Interestingly, regular training is an important prerequisite for maintaining cytokine levels and it has been shown that cytokine levels tend to return to their initial levels after 30 days without physical activity [40]. Remarkably, in a study by Qiu et al. (2014), the authors reported that regular exercise significantly lowers HbA1c concentrations in T2DM [41] as well as the systolic blood pressure after a 6-month exercise program [42]; however, they did not investigate the effects on periodontal health.

In both groups, the BMI did not change significantly during the study. We found an increase of 1.2 in the control group and a decrease of 1.1 in the intervention group. This may be a trend; however, there was no significant correlation to the groups. Possible changes in muscle to fat ratio were not analyzed. Independent of the BMI, systematic inflammation and glycemic control improved with periodontal healing.

From 108 participants, only 77 met the inclusion criteria. Unfortunately, there was also a high number of dropouts during the course of the study. Only 37 participants were examined over the whole period. The retention rate of the study was extremely low for the dental examinations. We believe that this was due to the extra time of 30 min that was necessary for the dental examination. Furthermore, the dental examination was scheduled at the end of the medical examinations which contributed to a reduced compliance of the participants. Consequently, in future studies, this should be addressed beforehand by special incentives to increase motivation for dental examinations. A limitation of the study is that we did not investigate oral hygiene behaviors at baseline and follow-up. It may be possible that participants were particularly motivated and that the health-promoting measures, such as eating habits or tooth brushing habits, were changed and may in part explain the positive effects observed.

## Conclusion

In summary, the results of the present study showed that physical activity is an oral health-promoting measure in patients with T2DM and physical activity significantly reduces HbA1c concentrations. Still, the lasting effect on oral health and the possible causal link remains unclear. In future clinical trials, it would be interesting to compare the treatment success of periodontal treatment with and without accompanying sports intervention.
